# Ethical frameworks should be applied to computational modelling of infectious disease interventions

**DOI:** 10.1371/journal.pcbi.1011933

**Published:** 2024-03-21

**Authors:** Cameron Zachreson, Julian Savulescu, Freya M. Shearer, Michael J. Plank, Simon Coghlan, Joel C. Miller, Kylie E. C. Ainslie, Nicholas Geard

**Affiliations:** 1 School of Computing and Information Systems, The University of Melbourne, Parkville, Victoria, Australia; 2 Centre for Biomedical Ethics, Yong Loo Lin School of Medicine, National University of Singapore, Singapore; 3 Biomedical Research Group, Murdoch Childrens Research Institute, Melbourne, Victoria, Australia; 4 Faculty of Philosophy, University of Oxford, Oxford, United Kingdom; 5 Infectious Disease Dynamics Unit, Centre for Epidemiology and Biostatistics, Melbourne School of Population and Global Health, The University of Melbourne, Parkville, Victoria, Australia; 6 School of Mathematics and Statistics, University of Canterbury, Christchurch, New Zealand; 7 Centre for AI and Digital Ethics, The University of Melbourne, Parkville, Victoria, Australia; 8 Department of Mathematical and Physical Sciences, La Trobe University, Bundoora, Australia; 9 Centre for Infectious Disease Control, National Institute for Public Health and the Environment, Bilthoven, the Netherlands; 10 School of Public Health, Li Ka Shing Faculty of Medicine, University of Hong Kong, Hong Kong Special Administrative Region, China; University of Washington, UNITED STATES

## Abstract

This perspective is part of an international effort to improve epidemiological models with the goal of reducing the unintended consequences of infectious disease interventions. The scenarios in which models are applied often involve difficult trade-offs that are well recognised in public health ethics. Unless these trade-offs are explicitly accounted for, models risk overlooking contested ethical choices and values, leading to an increased risk of unintended consequences. We argue that such risks could be reduced if modellers were more aware of ethical frameworks and had the capacity to explicitly account for the relevant values in their models. We propose that public health ethics can provide a conceptual foundation for developing this capacity. After reviewing relevant concepts in public health and clinical ethics, we discuss examples from the COVID-19 pandemic to illustrate the current separation between public health ethics and infectious disease modelling. We conclude by describing practical steps to build the capacity for ethically aware modelling. Developing this capacity constitutes a critical step towards ethical practice in computational modelling of public health interventions, which will require collaboration with experts on public health ethics, decision support, behavioural interventions, and social determinants of health, as well as direct consultation with communities and policy makers.

## 1. Introduction

The COVID-19 pandemic placed computational modelling of infectious disease into the international spotlight. The world has seen how models can inform the design of effective interventions, such as the lockdown policies that suppressed multiple pandemic waves through 2020 to 2022 in many parts of the world. However, even when interventions were effective, there were many negative consequences such as widespread disruption to food supply, substantial declines in rates of cancer screening, disproportionate impacts on disadvantaged ethnic groups and women, and increased incidence of psychological distress [1–5]. In some settings, policies were ineffective at mitigating the pandemic while also incurring severe human costs such as the internal displacement of migrant workers from urban centres during the national lockdown of India [6].

Because of the real-world consequences of public health interventions, the application of modelling to help guide policy to prevent or mitigate disease spread is not an ethically neutral activity. In general, modelling can be performed without considering ethics; however, when a model is developed to inform policy, its ethical implications should be accounted for. The intervention scenarios simulated by models often involve difficult trade-offs that are well recognised in public health ethics, such as liberty versus physical health, beneficence versus justice, and individual autonomy versus solidarity [7]. Unless these trade-offs are explicitly accounted for, models risk overlooking contested ethical choices and values, leading to an increased risk of unintended consequences.

We argue that such risks could be reduced if modellers were more aware of ethical frameworks and had the capacity to explicitly account for the relevant ethical values in their models. We propose that public health ethics can provide a conceptual foundation for developing this capacity. We begin by reviewing relevant concepts in public health and clinical ethics to demonstrate how ethical frameworks balance competing values such as physical health, outcome equity, and privacy. We then discuss examples from the COVID-19 pandemic to illustrate the current separation between public health ethics and infectious disease modelling. Finally, we conclude by describing practical steps towards ethically aware modelling practices.

## 2. Ethical frameworks in clinical practice and public health

Ethical frameworks are reasoning processes for translating intended moral outcomes into practical choices. They typically consider a set of values that may compete with one another and offer guidelines for balancing these trade-offs. For example, utilitarianism is a broad ethical framework according to which decided actions should maximise aggregate beneficial outcomes. Because utilitarianism has no intrinsic concern with the equitable distribution of goods and harms, this could require making some individuals or groups worse off than others. In this sense, it assigns exclusive value to beneficence (e.g., promoting physical and psychological well-being), quantified in aggregate [8]. By contrast, other frameworks of bioethics and public health ethics are more complex, often incorporating principles and values not only of beneficence or nonmaleficence (not perpetrating harms) but also of justice (i.e., equitable distribution of benefits and burdens) and respecting autonomy (e.g., civil liberties). Other principles and values include privacy, confidentiality, transparency, and accountability [9].

Comparing ethical frameworks in public health to those applied in the clinical context illustrates the difficulty of reconciling ethical principles across different social scales such as doctors’ offices, households, workplaces, cities, or states [10,11]. In clinical practice and research, in which the clinician–patient relationship is central and ethical frameworks have a longer history informing medical decision-making, autonomy and noninterference are often prioritised over outcomes related to physical health or healthy behavioural change (e.g., the principle of informed consent). Because an individual’s health decisions may affect the well-being of others, this tendency has led to practical and theoretical challenges associated with relational versus individualistic interpretations of autonomy [12,13].

While the decisions of individual patients in clinical settings may sometimes affect others, public health addresses scenarios in which the behaviour of individuals can influence the health of sizable segments of the population, as is the case during pandemics. In public health, interventions are designed by governments with the capacity for public information campaigns, large-scale incentive schemes, and ultimately the option of mandated interventions. The scope and scale of such interventions may result in approaches that prioritise values very differently from the clinical context. For example, public health may be more concerned with promoting large-scale beneficence and the equitable distribution of harms and benefits across the whole population, even when that substantially restricts liberty. An example is enforcement of non-pharmaceutical pandemic interventions that reduce disease transmission while seriously impinging on privacy and freedom of movement [14,15]. So, while similar values and principles apply to both clinical and population level settings, their prioritisation is typically quite different. The lack of a clear consensus about the ethical values and principles of public health responses, particularly regarding the definitions and boundaries of autonomy, makes it critical to adapt and define ethical frameworks on a case-by-case basis [16].

## 3. Ethics in pandemic modelling

The COVID-19 pandemic demonstrated the ethical trade-offs associated with infectious disease interventions. Polices such as lockdowns reduced disease spread but greatly infringed upon individual autonomy. Furthermore, such policies often imposed unequal burdens on specific population strata, such as families with children, and people who could not work from home. Meanwhile, imposition of digital contract tracing apps raised the ethical problem of balancing disease control with privacy [17]. Computational models were used to guide and justify these interventions throughout the pandemic, especially when uncertainty was high and decisions had to be made before transmission dynamics were well understood [18,19].

While this early modelling allowed a transparent and reproducible basis on which to establish assumptions and support decisions, and thus was arguably ethical in a broad sense, it was not fully consistent with modern frameworks of public health ethics. Below, we consider 3 broad categories of ethical values—autonomy, justice, and beneficence—and discuss some key ethical deficiencies of early COVID-19 modelling. We also provide examples of work that begins to address these issues:

**Autonomy:** Models typically only anticipated the direct behavioural response to government interventions (e.g., [20,21]), without considering the potential for spontaneous changes in behaviour to mitigate transmission (i.e., contact avoidance and discretionary isolation due to perceived infection risk). Implicitly, this simplification deprioritises values such as non-interference and physical liberty by devaluing the positive role autonomy can play in pandemic response. Later modelling efforts began to incorporate these features, sometimes influencing policy shifts away from mandated NPIs, as occurred in Denmark after emergence of the Omicron variant [22].

**Justice:** Optimisation of response measures to reduce aggregate clinical burden carries the risk of implicitly disregarding equity in the distribution of benefits and burdens, which is a prominent value in contemporary frameworks of public health ethics [23,24]. In general, short-term utilitarian objectives risk reinforcing structural inequity because lower costs are needed to prevent harms to individuals or groups who are more well-off in the first place [25]. Efforts are currently underway to quantify and address equity issues in epidemic modelling [26,27]. Additionally, at the intersection of Justice and Autonomy, recent work has introduced behavioural models that can account for inequities in discretionary behavioural response factors [28].

**Beneficence:** Some modelling reports acknowledged their limited capacity to account for values other than clinical burden (e.g., [19]). However, even in this respect pandemic intervention models had major deficiencies: Most models did not weigh different, potentially competing drivers of physical and mental health when simulating intervention effects. For example, few attempts were made to model impact on overall deaths, or excess morbidity [29]. Nor were effects on well-being generally modelled, which would be required by a fully utilitarian public health approach aiming to maximise aggregate benefit [8]. Promising approaches are being developed to address these deficiencies, including models of how the COVID-19 pandemic could impact the burden of other diseases [30].

Despite omitting consideration of broader public health values, the results of models can be compatible with modern ethical frameworks when they are balanced with other information. An integrated approach to decision-making can adapt to the diverse values of affected communities, for example, through collective reflective equilibrium in practice [31]. The narrow utilitarianism of the model would then act as a component in a more comprehensive balance of values produced by a collaborative process in which the ethical implications of model-based evidence are assessed post hoc, after model development. However, as the early COVID-19 response demonstrated, the time constraints of public health crisis situations may not allow for a nuanced cross-examination of model-generated evidence. In such cases, ethical deficiencies of a model may propagate implicitly into the decision framework guiding interventions.

We argue that if models were to *explicitly* incorporate ethical frameworks (however limited), they could play an important role in transparently evaluating the ethical trade-offs of potential interventions, moving beyond the post hoc ethical analysis described above. To illustrate this, we describe a hypothetical situation: Suppose a model compares 4 contact tracing systems: (a) no contact tracing; (b) traditional contact tracing based on voluntary phone interviews with health officials; (c) a voluntary app that logs contacts anonymously, with data stored on individual devices and shared with agencies only if someone tests positive; and (d) everyone required to carry a location-tracking device with data continuously transmitted to agencies. Suppose the model predicts decreasing public health burden from (a) to (d), but also quantifies the increasing amount and detail of personal information divulged to agencies. Whether the optimal trade-off is explicitly modelled or given to decision makers to determine, the model quantifies costs in terms of competing values. This allows a transparent choice on how to balance beneficence with privacy, while clearly delineating which values are considered in the model.

There are many practical hurdles to the development and application of models that can accommodate complex objective functions. To address equity concerns, models will require rich data streams to accurately capture the factors describing heterogeneous populations, and collecting accurate data carries its own ethical challenges [26]. Even if sufficient data were available, computational models have complexity limits and may not be able to quantify all important values simultaneously, especially when they relate to processes with different time scales (e.g., future morbidity due to decreased cancer screening during lockdown). Finally, a complex weighing of competing value trade-offs may need to be done independently of modelling. For example, knowledge about detailed real-world intervention scenarios can identify constraints on value maximisation that are based on complex contextual rules and social norms (deontological constraints) [8]. Rule-based constraints often correspond to large spaces of scenario permutations that are difficult to model. However, even when these challenges cannot be overcome, it is still ethically important to be explicit in the value objectives which drive a model, and to clearly delineate which values and rules it does not consider.

## 4. Implicit values from biased data

Value omissions are not the only means by which the implicit ethical frameworks in models may be insufficient. It is now understood that ethical risks arise when algorithms and models acquire biases from calibration data, with consequences documented in law enforcement, welfare allocation, and healthcare [32,33]. With respect to infectious disease modelling, this concept is discussed by Tizzoni and colleagues, who describe (e.g.) inequitable infectious disease surveillance arising from disparities in healthcare accessibility and healthcare-seeking behaviours [26]. Tizzoni and colleagues suggest incorporation of socioeconomic strata as core model design features. Such techniques will be critical to modelling equitable public health interventions, but should be developed carefully: An ethically aware modelling approach will recognise the risk of stigmatisation and that historical inequities may propagate implicitly into model-generated evidence through biased data streams describing disease burden, risk factors, and behavioural trends [26].

## 5. Practical considerations for modellers

As an ongoing process, actionable in the short term, we advocate for the expansion of ethical literacy in the modelling community. Even without changing technical aspects of existing models, literacy will allow productive dialogue between decision makers and modellers about the ethical implications of model features and assumptions. Such dialogue can facilitate progress towards next-generation models by identifying opportunities for technical advancements that address the ethical deficiencies of current approaches.

Ultimately, modellers should not be limited to post hoc evaluation of a model’s ethical implications. Rather, ethical frameworks should act as development guidelines. Using ethical frameworks as core principles of policy-relevant model design means:

1. understanding the set of ethical values that informs the policy objectives,
2. choosing modelling techniques that are compatible with that set of values,
3. evaluating how the values are specifiable in the implemented model.

**Fig 1:**
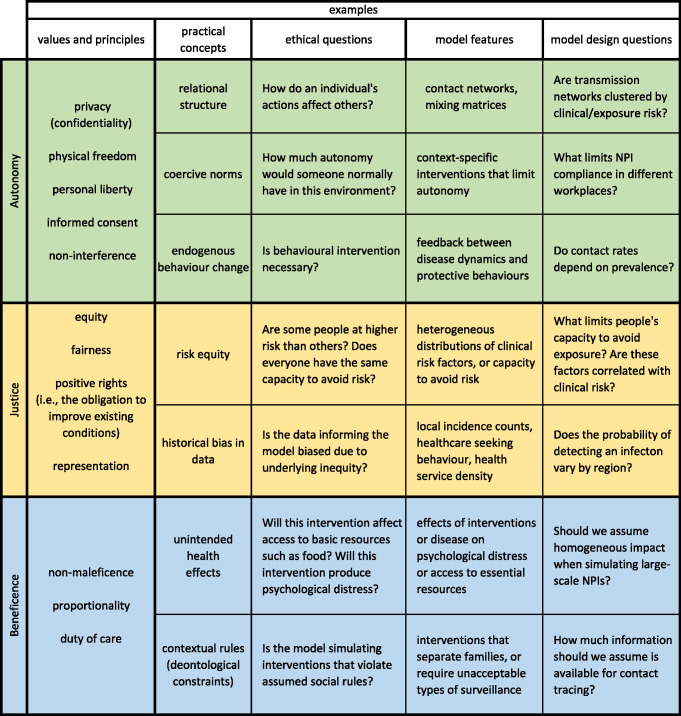
Examples of practical guidelines for identifying or incorporating ethical values in computational models of infectious disease interventions. Colors correspond to broad categories of values ubiquitous in frameworks of public health and clinical ethics. For each, representative lists of subordinate values and principles are joined to practical concepts that could inform intervention policy, ethical questions arising from the associated practical concepts, model features necessary for accommodating the practical concepts, and model design questions arising from the associated ethical questions and values.

These 3 elements will allow iterative improvement to make models more ethically aware. We do not suggest that prioritising values and specifying the ethical frameworks for policy decisions should be the responsibility of model developers. Rather, developers should be able to implement models that can compare potential decisions within a specified ethical framework, as established through collaboration and consultation. While developing a comprehensive strategy for implementing the above steps is beyond the scope of this perspective, Fig 1 lists some (non-exhaustive) guidelines for ethically aware modelling organised by value categories. Currently, the importance of elements like these is recognised by the modelling community, and addressing the limitations of current approaches is the subject of ongoing research [26–28,34].

## 6. Conclusion

Largely due to the COVID-19 pandemic, there are now thousands of unique models of infectious disease transmission that simulate public health interventions. These models incorporate implicit assumptions about human behaviour and imply sets of values and priorities upon which public health decision makers should operate. Such assumptions imply ethical frameworks that define the prioritisation of values and outcomes such as reducing public health burden, the equitable distribution of benefits and burdens, and personal autonomy. Modellers should develop the capacity for these frameworks to be made explicit, enabling models that integrate well-specified ethical frameworks as core design elements. Developing this capacity will empower modelling practitioners to define and understand the values embedded in models. However, modellers do not, and should not, work in a vacuum. Incorporating ethics into computational modelling of public health interventions will require collaboration with experts on public health ethics, decision support [35], behavioural interventions [36], and social determinants of health [37]. This will position the modelling community to more effectively support ethical decision-making, through direct consultation with communities and policy makers. Building ethical awareness in the modelling community will lead to increased trust that the models used in decision support reflect the balance of values held by affected communities, reducing the impact of unintended consequences, and making the associated policies more likely to succeed.
